# Research on engine power-loss fault diagnosis method based on time-series data mining

**DOI:** 10.1038/s41598-025-32777-2

**Published:** 2025-12-16

**Authors:** Li Feng, Le Liu, Hongsheng Xu, Sumeng Gao, Hai Tang, Jianing Cui, Bin Zhang, Yufeng Rao

**Affiliations:** 1 School of Intelligent Connected Vehicle, Hubei University of Automotive Technology, Shiyan, 442002 China; 2 Hubei Provincial Engineering (Technology) Research Center of Automotive Intelligent Networking and Electronic Control, Hubei University of Automotive Technology, Shiyan, 442002 China; 3 International Joint Research Center of Automotive Cloud Computing and Simulation Control, Hubei University of Automotive Technology, Shiyan, 442002 China

**Keywords:** Time-series data mining, Engine power-loss fault, Diagnostic approach, Commercial vehicles, Machine learning, Deep learning, Energy science and technology, Engineering, Mathematics and computing

## Abstract

Traditional diagnostic approaches for engine power-loss faults in commercial vehicles are limited by their heavy reliance on on-site road testing and high consumption of human and material resources. To address these limitations, this study proposed a new intelligent diagnosis method based on time-series data mining. Analyzing real-world operational data collected from onboard telematics terminals, identified key features strongly correlated with engine power loss, including vehicle speed, acceleration, and the rate of change of throttle opening. Building upon these features, a dual-framework diagnostic strategy was developed: the data were first categorized into two groups, “with driver acceleration intent” and “without driver acceleration intent”, based on the rate of change of throttle opening. For samples with acceleration intent, multiple machine learning algorithms were employed to model and diagnose vehicle power performance; for those without driver acceleration intent, a deep learning model integrated with classification techniques was introduced to detect latent power-loss faults. Experimental results demonstrated that the proposed method achieved high accuracy and specificity in fault identification, confirming its effectiveness and practical potential. This study provides a viable pathway toward remote, online diagnosis of engine power loss in commercial vehicles and lays a foundational framework for the intelligent advancement of this field.

## Introduction

Engine faults account for roughly 40% of total vehicle malfunctions^1^, with engine power insufficiency being a common issue that is often hard to detect and prevent. This type of fault manifests as inadequate vehicle power, leading to reduced power and torque, increased fuel consumption, higher costs, and greater environmental impact. It also compromises driving stability and safety. Prolonged power insufficiency can accelerate engine and component wear, shortening the vehicle’s lifespan. Accurate diagnosis and maintenance of vehicle power performance are therefore crucial. Traditionally, diagnosing engine power insufficiency has relied on onsite test-driving. Experts compare the acceleration and speed of suspected faulty vehicles with those of normal vehicles under full-throttle conditions. This method depends on expert experience and an understanding of fault mechanisms. It is inefficient, has vague standards, and is resource-intensive. Some scholars have proposed using advanced technologies and intelligent systems to assist or replace traditional manual judgment^2,3^. With the rapid development of sensor monitoring, communication technologies, big data analytics, and artificial intelligence, data-driven methods have become a new trend in the field of intelligent fault diagnosis. A novel anomaly detection model recommendation method was proposed in^4^, which uses a data-driven approach to detect anomalies in time-series data. Meanwhile, the intelligent fault diagnosis (IFD) method based on domain generalization (DG) has been effectively applied in^5^ to address the challenges brought by domain shifts when facing unseen target domains. In addition, multi-source domain generalization (DG) has also made significant progress in cross-speed fault diagnosis^6^. The application of attention mechanisms also provides a boost to the diagnostic methods, Recently, to address the challenge of comprehensively capturing fault information from multi-sensor signals in large rotating machinery, one study proposed a multi-sensor multi-head graph attention network (MMHGAT)^7^ framework that enables end-to-end high-level feature extraction through dual graph attention layers and a dynamic feature fusion mechanism. Another work^8^ introduced a scale attention mechanism that adaptively selects the optimal neighborhood aggregation scope to effectively integrate multi-scale graph structural information, thereby enhancing node-level classification performance. Furthermore, to tackle the issue of inconsistent signal quality across channels under noisy conditions, a multiscale channel attention-driven graph dynamic fusion network was developed^9^, which computes channel attention weights at multiple scales to strengthen node representations and significantly improve diagnostic robustness. These methods achieve equipment abnormality monitoring and fault diagnosis by conducting in-depth analysis and mining of the collected data, revealing the intrinsic connections between the current operating status and historical records. Compared with traditional methods, data-driven strategies do not rely on prior knowledge about the automotive engine fault system, thereby realizing the automation of the fault diagnosis process^10–12^. This shift not only enhances the efficiency and accuracy of fault diagnosis but also paves new research directions and technological pathways for intelligent fault diagnosis.

In current academic research, vehicle-data-based fault diagnosis is still in its early stages, especially regarding engine power performance. Most studies only improve on traditional methods. To make power-performance evaluation more accurate and practical, this study focuses on a commercial-vehicle company’s engine. It uses Internet of Vehicles technology to collect real-world driving data instead of relying on onsite test-driving data. Combining traditional fault-diagnosis methods and data-mining techniques, it proposes a new engine power-loss fault diagnosis scheme from a time-series-data perspective.

To address the issue of partial vehicle data lacking explicit acceleration intention and to construct a comprehensive diagnostic model, an initial assessment of vehicle data is required to determine the presence of acceleration intention. Based on whether the driver’s acceleration intention exists, the data is divided into two categories for processing. For data with driver acceleration intention, a machine learning model is employed for diagnosis, as it excels in handling data with clear features and smaller datasets, enabling rapid and accurate diagnosis. In contrast, for data without clear driver acceleration intention, a deep learning model is utilized, leveraging its ability to automatically extract features and effectively diagnose complex, ambiguous, and larger datasets. This research is structured around methods and experiments, and concludes with a discussion and summary, providing a solid foundation for the implementation of online diagnosis.

### The work of this paper is as follows

This work reports a methodology and results that are in the following manners:

1. Extract the refined feature of driver acceleration intention from the concept of driving intention, and introduce it as a sampling method into the diagnosis of engine power failure.
2. For different acceleration intent scenarios of various drivers, diagnostic models are constructed using traditional machine learning methods to achieve precise diagnostic results.
3. For data lacking acceleration intent, a diagnostic framework based on Transformer is established, incorporating the new xLSTM algorithm module, and necessary tuning is performed to achieve the identification of faulty samples.

## Materials and methods

###  Scoping review

 In industrial time-series data prediction, a key challenge in fault diagnosis is capturing the dynamic changes in equipment status from time-series data. Historical data from sensors is often used to train models for various applications. Early time-series forecasting techniques mainly relied on statistical and mathematical models. Models like SARIMA^13,14^ and TBATS^15^ analyze trends, seasonality, and periodicity in historical data to make predictions. While effective in some scenarios, these traditional models have limitations in handling complex nonlinear relationships and long-term dependencies. With the rise of deep-learning technologies, many scholars have shifted their focus to this area. RNN^16^, one of the early deep-learning models, have been widely used in time-series forecasting. However, they are prone to gradient explosion and vanishing problems during training, limiting their application in long-sequence modeling. To overcome these issues, improved RNN such as LSTM^17^ and GRU have emerged. These models use special gating mechanisms to better capture sequential dependencies and long-term patterns in data^18^. LSTM are particularly suitable for time-series data. Their unique cell state and gating structure enable stable processing of long sequences and effective learning of key data features. In addition to RNN^19^, CNN^20^ have also shown good performance in time-series forecasting. CNN automatically extract local features through convolution operations and can effectively capture local patterns and trends in time-series data. In recent years, the Transformer^21^ architecture has achieved great success in natural language processing and has been applied to other fields such as image processing, speech recognition, and time-series forecasting. The core of the Transformer is the self-attention mechanism, which dynamically focuses on different data positions to capture global dependencies. This makes Transformers potentially advantageous for handling time-series data with complex dependency structures.

###  Data acquisition

This study investigates the power deficiency issue in commercial vehicles produced by a specific manufacturer. Utilizing real-world operational maintenance data from DDi11E465-60 engine-equipped commercial vehicles, experimental tests were conducted to validate the effectiveness of the proposed diagnostic model. The dataset encompasses historical operational data from 20 vehicles over a two-month period. These vehicles exhibited engine power deficiency symptoms and were subsequently sent for repairs at a specific time point, with post-repair diagnostics confirming engine fault resolution. The repaired vehicles resumed normal operation within 24 h. For analysis, the five-day operational data collected prior to fault diagnosis(hereafter termed “faulty phase data”) and the five-day data recorded immediately following repair(termed “normal phase data”) were systematically compared. The data comes from real data provided by a commercial vehicle, with telematics collecting high-frequency (over 1 Hz) signals. Given that commercial vehicles typically operate up to 10 h daily, the substantial volume of data offers a detailed reflection of the vehicle’s dynamic behavior.

###  Data processing

Based on expertise from engine specialists, features related to engine power loss were selected from the vehicle terminal data, providing a theoretical basis for fault modeling. A preprocessing and refinement methodology was developed for diagnosing engine power loss using real driving data. Initially, 74 features were extracted-including vehicle speed, throttle position, engine speed, and coolant temperature. To mitigate redundancy and enhance model accuracy and efficiency, the most representative features were selected and further derived. Research indicates that when a driver intends to accelerate, changes in the vehicle’s power performance can be accurately captured^22^. And that a driver’s acceleration intent can be inferred from changes in power performance metrics. In particular, the rate of change in throttle position reveals underlying control intentions. By quantifying and classifying this rate, the vehicle’s power state can be evaluated, forming an acceleration-intent-centered dataset.

Literature review and real-world data analysis^23^ confirm that acceleration reflects power performance, with throttle position being a key control variable. However, throttle position alone is insufficient to determine acceleration intent-a high value may not indicate urgent acceleration, whereas a low value might still reflect moderate intent. Thus, both throttle position and its change rate were adopted as parameters. After analysis, the following key features were identified: initial and final speed, duration, average acceleration, initial/final/average throttle position, and positive throttle change rate.

To ensure data quality, missing and abnormal values were removed based on normal driving patterns. For consistency with traditional test methods, segments were selected from the same vehicle model, on flat roads, with identical gear-shift behavior.

The throttle change rate peaks around 0.1s after movement initiation, with acceleration peaking approximately 1.2s later. To avoid delay artifacts, continuous data segments starting from throttle onset are extracted. Segment duration is determined via acceleration clustering to distinguish normal and faulty states. Most throttle change rates fall between 40% and 70%. Values below 40% indicate mild acceleration with limited variation and are excluded. driver acceleration intents are classified as mild, moderate, or rapid to improve analytical granularity. Stable segments within the 40–50%, 50–60%, and 60–70% intervals are selected to ensure reliability. Full-throttle performance is a key indicator of maximum power output. Real-world data, collected via vehicle-network technology and validated by engine experts, ensures that selected acceleration segments align with on-site test standards, enhancing practical relevance and credibility.

####  Experimental protocol

**Fig. 1:**
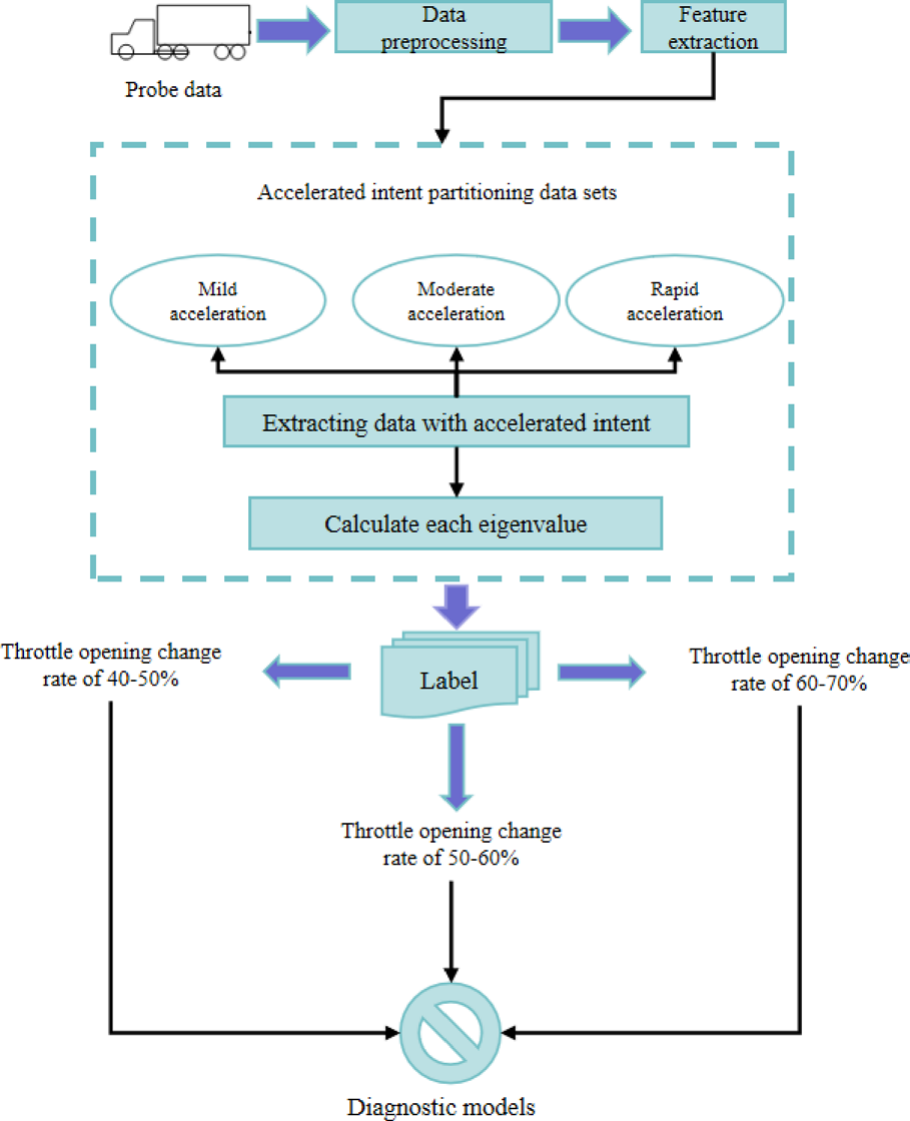
The diagnostic framework based on driver acceleration intent.

For the data with driver acceleration intent: As shown in Fig. 1, the diagnostic framework for engine power loss fault based on driver acceleration intent comprises data preprocessing, feature extraction, driver acceleration intent classification, and fault identification. Firstly, screen the main features related to power performance from the data collected by vehicle terminals and perform data preprocessing. Then, extract driving data with driver acceleration intent based on throttle behavior, and following expert discussions, classify it into mild (throttle position change rate of 40%-50%), moderate (50%-60%), and rapid acceleration (60%-70%)^24,25^. Next, for each throttle driver acceleration intent, extract corresponding data segments and compute the variables required for the experiment. Over a certain time length, construct a dataset by labeling the features of normal and faulty data. Finally, train the selected model on historical data to enable it to recognize data features under normal conditions and determine whether there is an engine power-loss fault.

**Fig. 2:**
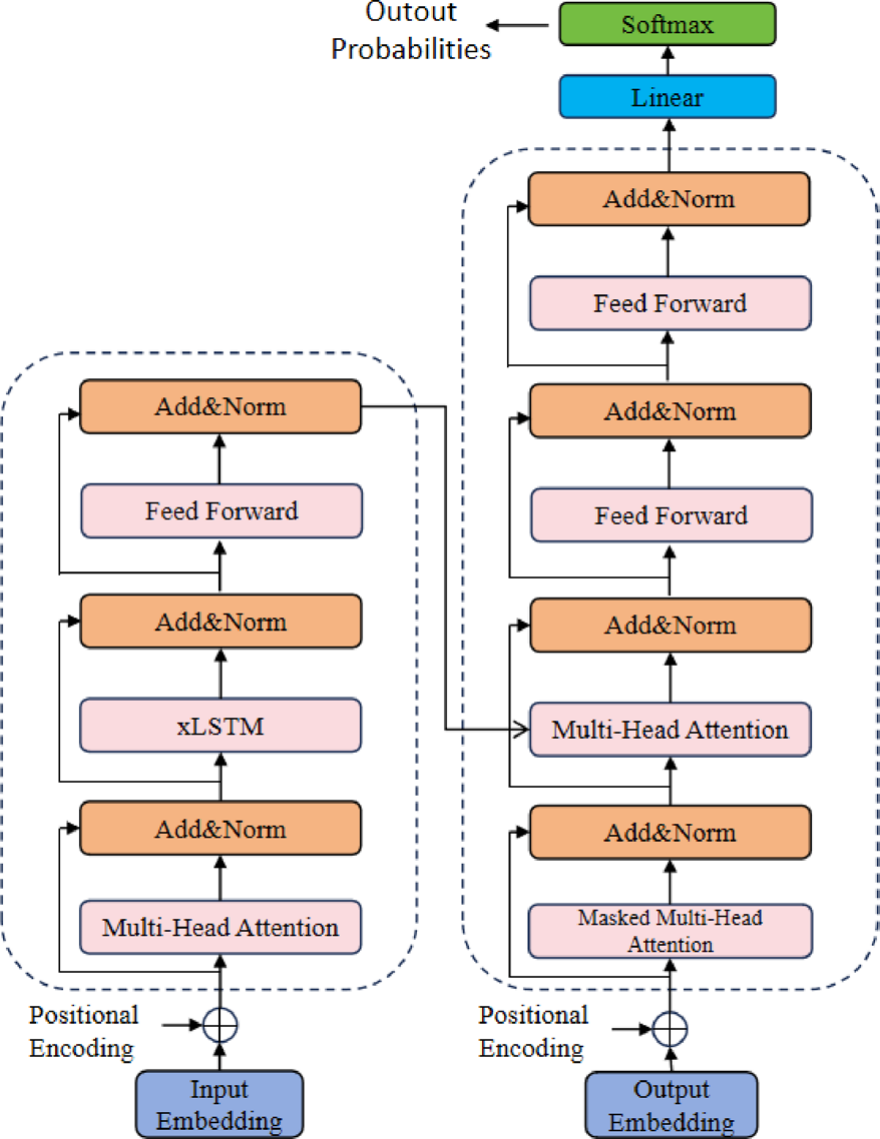
The framework diagram for diagnosis without driver acceleration intent.

For the data without obvious driver acceleration intent: Raw time-series data is preprocessed using a sliding window to capture local pattern features in continuous time series. Then, a core diagnostic model is built with the Transformer framework to capture global relationships using its self-attention mechanism. To meet the long-term dependency modeling needs of time-series data, an xLSTM^26^ module is embedded in the Transformer encoder to strengthen the capture of long-span dependencies through gating mechanisms. Also, an extra feed-forward neural network (FFN) layer is added after the standard Transformer decoder to enhance the model’s complex-feature representation through nonlinear transformations, addressing the limitations of the single self-attention mechanism in feature fusion. The specific model is shown in Fig. 2.

### Experimental model

#### Driver acceleration intent diagnosis model

The engine power-loss fault diagnosis is treated as a binary-classification problem. To boost diagnostic accuracy, reliability, and model adaptability, different driver acceleration intents are addressed with distinct methods. KNN determines class based on neighboring samples, making it suitable for datasets with overlapping classes like mild and moderate acceleration. SVM, capable of handling small samples and finding optimal hyperplanes in high-dimensional spaces, is used for rapid-acceleration data.

#### Without obvious driver acceleration intent diagnosis model

In fault diagnosis, extracting nonlinear features and modeling long-distance dependencies in non-stationary time-series data are two key challenges. To address the monitoring of engine power performance in commercial vehicles without driver acceleration intent, this paper proposes an intelligent diagnostic method that integrates xLSTM with a hierarchical Transformer architecture. The model takes multi-dimensional time-series signals, sampled via a sliding window, as input, and performs end-to-end classification through an embedding transformation, an encoder-decoder architecture, and a classification layer. The following provides a formal mathematical description of the model.

##### Input representation and embedding layer

Let the input time-series data be denoted as:

1 $$X=[{x_1},{x_2},...,{x_T}] \in {R^{B \times T \times D}}$$

Where *B* is the batch size, *T* is the sequence length, and *D* is the number of features per time step.Each input vector *x*_*t*_ is first projected into a higher-dimensional space via a linear embedding layer:

2 $$E_{t} = W_{e} x_{t} + b_{e} ,t = 1,2,...,T$$

3 $${W_e} \in {R^{{d_{\bmod el}} \times D}}, {b_e} \in {R^{{d_{\bmod el}}}}$$

4 $$E={[{E_1},{E_2},...,{E_T}]^T} \in {R^{B \times T \times {d_{\bmod el}}}}$$

Where (3) are learnable parameters, and *d*_*model*_ is the embedding dimension. The resulting embedded sequence is (4), which serves as the input to subsequent layers.

##### xLSTM-augmented transformer encoder

The encoder consists of *N =* 4 stacked encoder blocks. Each block includes multi-head self-attention, an xLSTM layer, a feed-forward network (FFN), and residual connections with layer normalization. Multi-Head Self-Attention.

Multi-Head Self-Attention: Given the input *E*, query (Q), key (K), and value (V) matrices are computed as:

5 $$Q=E{W^Q},K=E{W^K},V=E{W^V}$$

6 $${W^Q},{W^K} \in {R^{{d_{\bmod el}} \times {d_v}}}$$

Where (6) with *d*_*k*_*=d*_*v*_*=d*_*model*_*/h*, and *h =* 2 being the number of attention heads. The scaled dot-product attention for each head is defined as:

7 $$Attn(Q,K,V)=soft\hbox{max} (\frac{{Q{K^T}}}{{\sqrt {{d_k}} }})V$$

The outputs of all heads are concatenated and linearly transformed:

8 $$MHA(E)=Concat(hea{d_1},...,hea{d_h}){W^O}$$

xLSTM Feature Enhancement: Following the self-attention module, an xLSTM layer is applied to enhance modeling of long-term dependencies and nonlinear dynamics. *Let A = MHA(E)*, then:

9 $$H,S=xLSTM(A)$$

10 $$H \in {R^{T \times B \times {d_{\bmod el}}}}$$

Where (10) is the output hidden state, and S represents internal memory states. In this work, the xLSTM adopts a multiplicative LSTM (mLSTM) structure^27^, enabling input-dependent modulation of gate activations for improved feature selection.

Residual Connection and Layer Normalization: The output is refined via residual connection and layer normalization:

11 $${Z^{(1)}}=L{\mathrm{ay}}erNorm(E+Dropout(H))$$

Feed-Forward Network (FFN): A position-wise FFN applies nonlinear transformation:

12 $$F={W_2}\cdot ReLU({W_1}{Z^{(1)}}+{b_1})+{b_2}$$

Where *W*_*1*_∈*R*^*dff×dmodel*^, *W*_*2*_∈*R*^*dmodel×dff*^ and *d*_*ff*_=16. Another residual connection yields:

13 $${Z^{(2)}}=L{\mathrm{ay}}erNorm({Z^{(1)}}+Dropout(F))$$

This process is repeated N times, producing the final encoded representation *E*_*out*_∈*R*^*T*×*B*×*dmodel*^.

##### Transformer decoder

The decoder also comprises *N* = 4 identical layers, each containing three sub-layers:

Masked Multi-Head Self-Attention: Prevents information leakage from future time steps;

Encoder-Decoder Cross-Attention: Uses *E*_*out*_ as Key and Value;

Position-wise Feed-Forward Network: Let the initial decoder input be *D* = *E* (shared embedding). The output of the *l*-th decoder layer is:

14 $${D^{(l)}}=DecoderBloc{k^{(l)}}({D^{(L - 1)}},{E_{out}})$$

The final decoder output is denoted as

15 $${D_{out}}={D^{(N)}} \in {R^{T \times B \times {d_{model}}}}$$

## Experiments and results

### Driver acceleration intent diagnosis

To determine the optimal data-segment duration, a series of experiments were conducted. These experiments focused on data segments lasting from four to eight seconds. The results indicated that data segments of six seconds in length provided the highest accuracy. This was because, on the one hand, it avoided the problem of information dilution that can occur with longer data segments. On the other hand, it ensured the reliability and integrity of the data. Therefore, a six-second duration was established as the standard for data segments. The distribution of specific sample data is shown in Table 1.

Data for the experiment, aimed at building a diagnostic model for engine power loss across different driver acceleration intents, was collected from a vehicular communication platform. Data from vehicle repairs was carefully selected, with data from five days prior to the repair serving as the fault dataset and data from five days after the repair as the normal dataset. The normal data group is marked as 1, and the fault is marked as 0.

**Table 1:** Accelerate intent classification data sets.

Driver acceleration intents	Change rate of throttle opening	Normal/Group	Fault/group
Mild acceleration	40%~50%	736	254
Moderate acceleration	50%~60%	355	109
Rapid acceleration	60%~70%	48	15

#### Mild and moderate acceleration intent

Experiments comparing RNN, BP Neural Networks, SVM, and KNN were conducted. In this study, the KNN algorithm utilized a grid search approach to identify the optimal number of neighbors within the range [1, 3, 5, …, 19, 21], adopting the Euclidean distance metric and uniform weighting. The analysis concluded that an optimal neighbor count of 5 yielded the best performance. The BP Neural Network was constructed with a two-hidden-layer architecture. Parameter optimization was conducted through random search, focusing on the number of neurons in the first hidden layer [64,128], the second hidden layer [32,64], learning rates sampled from a log-uniform distribution between [0.001,0.01], and dropout rates ranging from [0.2 to 0.5]. The Adam optimizer was selected for model training. Following optimization, the final network configuration consisted of 128 and 64 neurons in the first and second hidden layers, respectively, a learning rate of 0.001, and a dropout rate of 0.3. SVM models were optimized using grid search methods for both linear and Radial Basis Function (RBF) kernels. For the linear kernel, the penalty parameter C was tuned across the values [0.1,1,10], whereas for the RBF kernel, both C [0.1,1,10,100] and gamma [0.01,0.1,1] were adjusted. The findings indicated that the RBF kernel with C = 10 and gamma = 0.1 achieved superior performance compared to other configurations. Regarding the RNN, a single-layer architecture was employed, with Bayesian optimization applied over 50 trials to determine the optimal hidden layer dimensions [64,128,256] and dropout rates [0.2,0.4,0.6]. The Adam optimizer was used with a fixed learning rate of 0.001. Optimization results showed that a hidden layer size of 128 and a dropout rate of 0.4 provided the most effective model configuration.

**Fig. 3:**
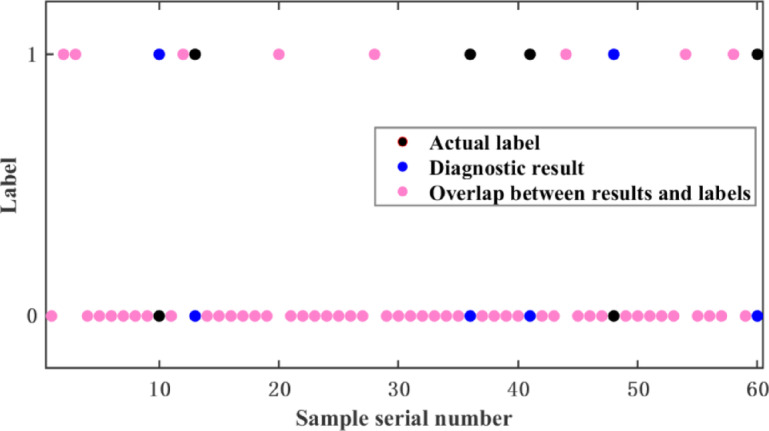
mild acceleration classification renderings.

**Fig. 4:**
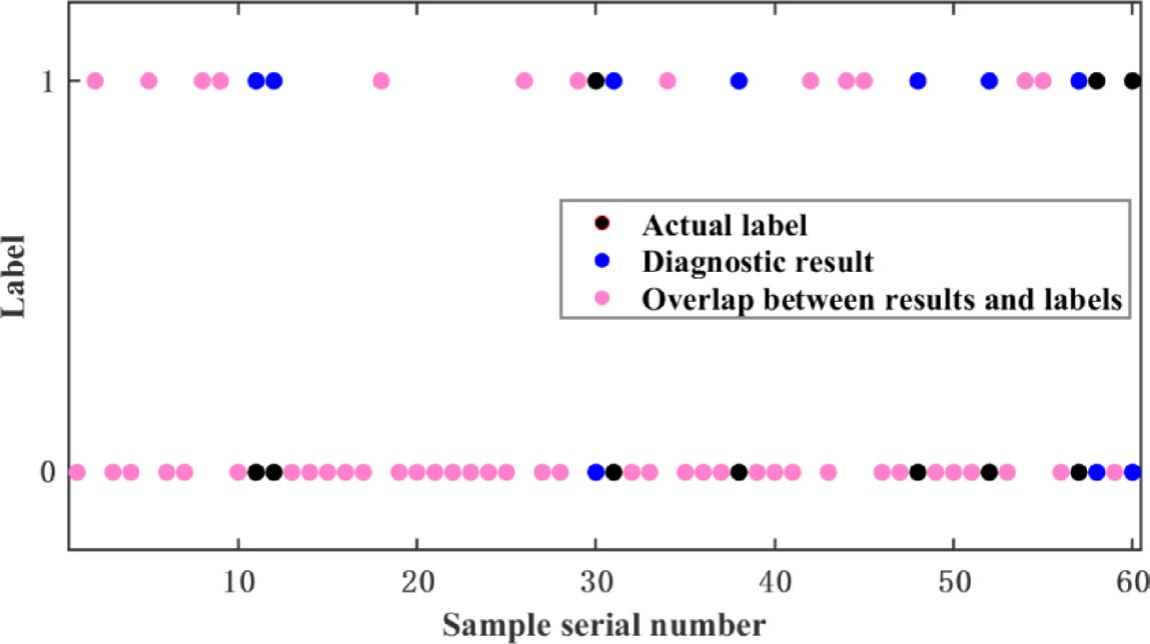
moderate acceleration classification renderings.

The KNN algorithm was used to train the model. As shown in Figs. 3 and 4, the classification results of the first 60 validation-set data under mild and moderate driver acceleration intents are presented. Pink markers indicate correct predictions. Pink markers’ proportion shows the model’s accuracy in predicting different power states, offering an intuitive evaluation and optimization basis. The y-axis represents normal power (0) and power insufficiency (1). Tables 2 and 3 show the results. KNN outperformed traditional machine-learning methods in diagnosing mild and moderate driver acceleration intents, with accuracies of 86.1% and 91.6%. It better captures both local and global data structures, reducing overfitting and enhancing prediction reliability.

**Table 2:** Comparison of the performance of the mild acceleration model.

Model	Accuracy	Recall	Precision	F1-score
BP neural networks	0.791	0.791	0.835	0.812
RNN	0.768	0.768	0.710	0.737
SVM	0.821	0.859	0.662	0.747
KNN	0.861	0.849	0.873	0.861

**Table 3:** Comparison of the performance of the moderate acceleration model.

Model	Accuracy	Recall	Precision	F1-score
BP neural networks	0.843	0.843	0.861	0.852
RNN	0.871	0.871	0.896	0.883
SVM	0.849	0.917	0.682	0.782
KNN	0.916	0.921	0.919	0.919

#### Rapid acceleration

The dataset for rapid acceleration intent is relatively small but closely mirrors traditional test-driving methods, ensuring reliability and effectiveness. Therefore, the SVM was chosen to build the diagnostic model for engine power insufficiency.

**Fig. 5:**
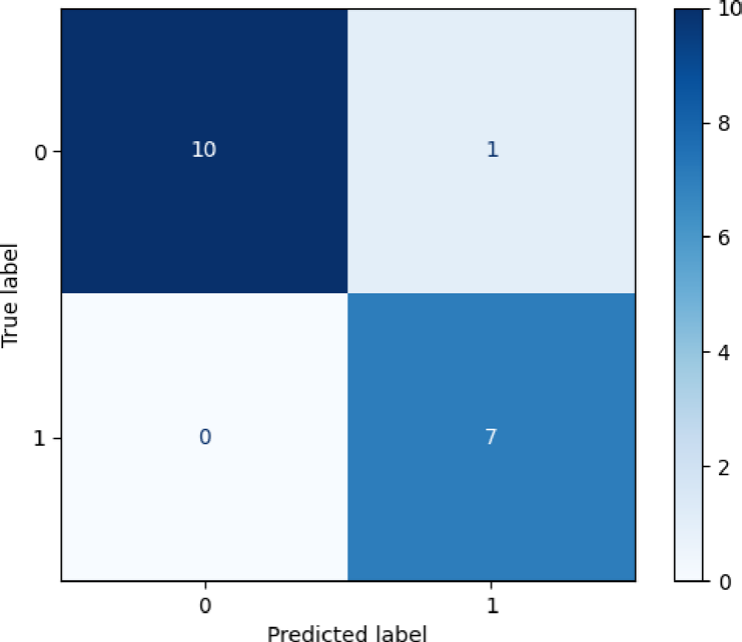
Confusion matrix of the classification results using the SVM MODEL.

To gain a comprehensive understanding of the experimental results, a confusion matrix was used for statistical analysis, as shown in Fig. 5. It indicates that among 18 test samples, only one normal vehicle was misdiagnosed as faulty, while all faulty vehicles were correctly identified. This demonstrates the model’s high accuracy and reliability in fault detection.

Compared to BP Neural Network, RNN, and KNN, SVM shows remarkable diagnostic performance in rapid acceleration conditions with limited samples. As shown in Table 4, SVM achieves 94.4% accuracy, effectively identifying vehicle power insufficiency. Its recall rate of 100% means all faulty vehicles are detected, reducing false negatives. Additionally, the F1-score of 93.3% indicates a well-balanced precision and recall, reflecting high-quality diagnostic results.

**Table 4:** Comparison of the performance of the rapid acceleration model.

Model	Accuracy	Recall	Precision	F1-score
BP neural networks	0.794	0.739	0.646	0.689
RNN	0.833	0.833	0.798	0.815
KNN	0.895	0.895	0.862	0.878
SVM	0.944	1.000	0.875	0.933

#### Accelerated intention comparison experiment without division

To verify the effectiveness of acceleration-intention categorization, we compared scenarios with and without it using collected data. As shown in Table 5, the classification accuracy was 72.8% without intention categorization. After categorization, accuracy improved across all cases, and it increased with the intensity of driver acceleration intent. This confirms the validity of categorizing driver acceleration intent by intensity.

### Without obvious driver acceleration intent diagnosis

**Table 5:** Comparison of different acceleration Intents.

Model	Accuracy	Recall	Precision	F1-score
Accelerated intention without division	0.728	0.728	0.668	0.697
Mild acceleration	0.861	0.849	0.873	0.861
Moderate acceleration	0.916	0.921	0.919	0.919
Rapid acceleration	0.944	1.000	0.875	0.933

To verify the effectiveness of Transformer, xLSTM, and the sliding window mechanism in fault diagnosis of time-series data, a series of experiments were designed. The experimental dataset comprises multi-feature operational data, Among them, after sampling using a sliding window, the dataset contains 3,000 fault samples and 3,000 normal samples. All models were trained on the same training set, fine-tuned on the validation set, and finally evaluated on the test set. To improve prediction accuracy and reduce the impact of non-standard data, standardize all the data.

#### Parameter setting

During the experiment, we considered the impact of sliding-window size on results. To find the optimal size accurately, we conducted experiments focusing on how different window sizes (5–20) affect model performance. We found that a window size of 10 maximizes model accuracy. This size ideally balances preventing information sparsity and ensuring data quality by avoiding overly long windows while maintaining data reliability and integrity. Thus, we set 10 as the standard window size. Subsequently, we transformed the data into a sequence format, assigning a unique label to each window to provide structured data for model training.

Model parameters were set as follows: embedding dimension of 8, 16 hidden-layer neurons, 2 multi-head attention heads, dropout rate of 0.1, and 4 layers each for the encoder and decoder. The learning rate was 0.0005, and the L2 regularization coefficient was 1e-4.

The preprocessed dataset was split into training, validation, and test sets in a 7:1:2 ratio. DataLoaders were used to facilitate batch data loading for training and evaluation.

During training, a weighted cross-entropy loss function was applied to address class imbalance by assigning class-specific weights. The Adam optimizer was used with a StepLR scheduler to halve the learning rate every 10 epochs, accelerating convergence. After each training epoch, training and validation losses were recorded for visualization. Early stopping was triggered if the validation loss remained unchanged for 10 epochs to prevent overfitting.

#### Experiment and analysis

**Fig. 6:**
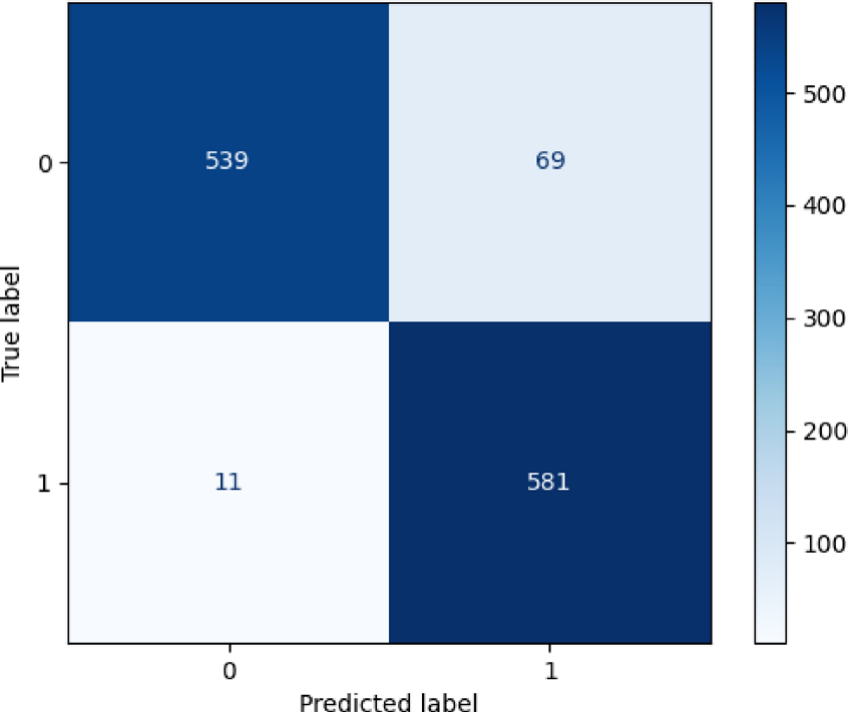
Confusion matrix of classification results.

**Fig. 7:**
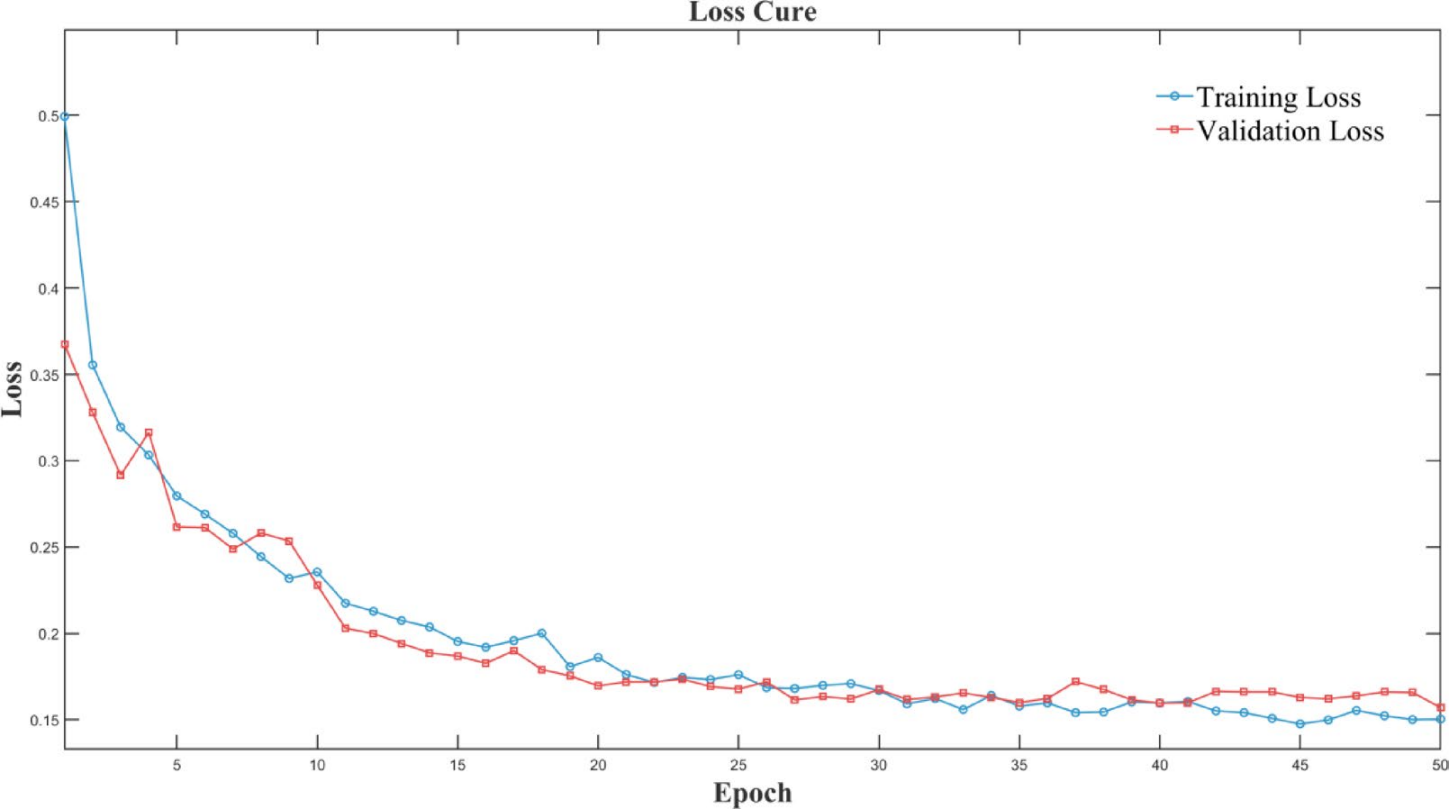
Model loss function iterative process.

**Fig. 8:**
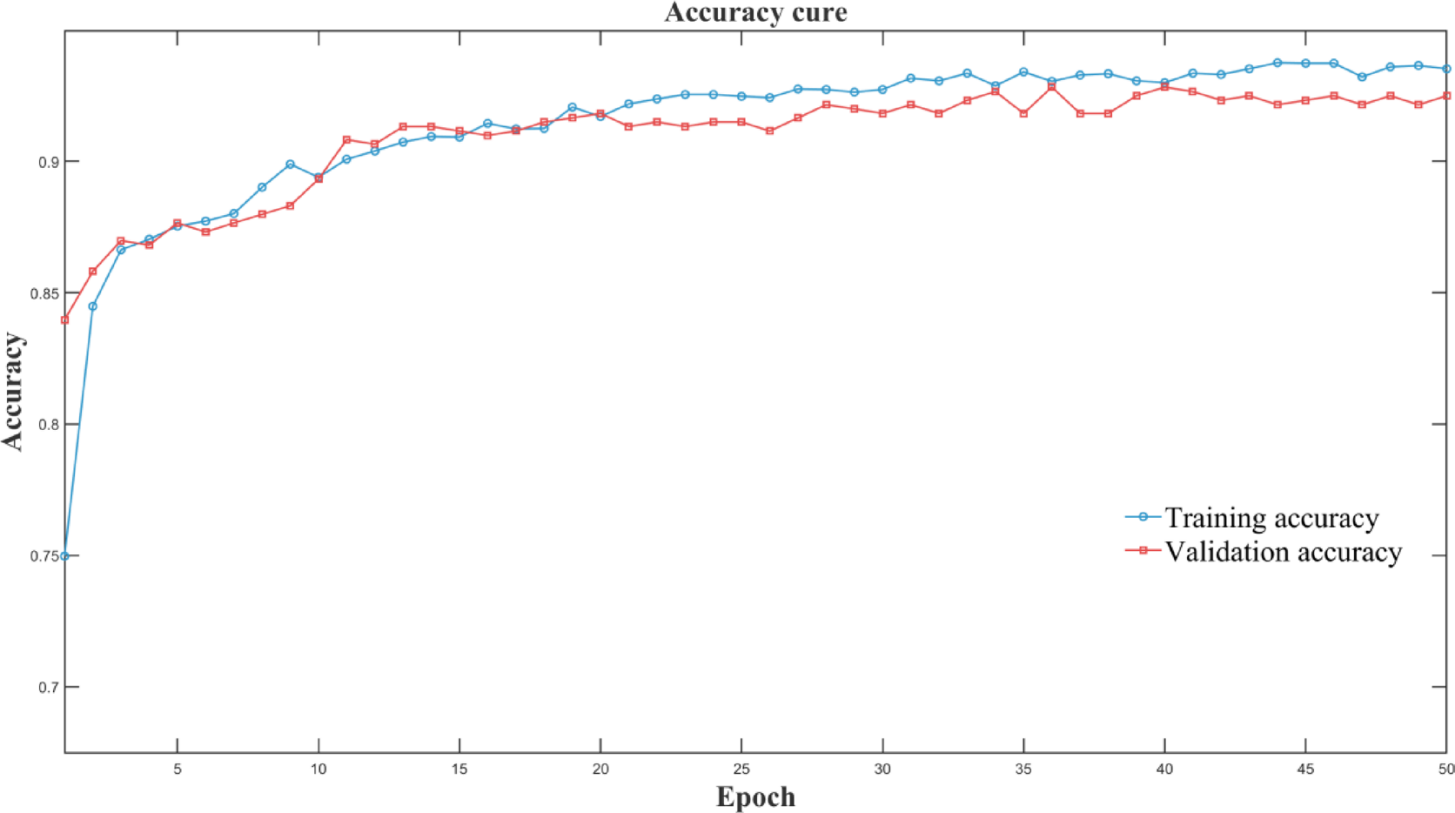
Model accuracy rate iterative process.

To gain a comprehensive understanding of the experimental results, a statistical analysis was conducted using a confusion matrix and loss-function plots, as shown in Figs. 6, 7 and 8.

The Figs. 6 confusion matrix shows that in 1,200 test samples, only 11 normal vehicles were misdiagnosed as faulty ones, and faulty vehicles were highly recognizable. The Fig. 7 loss-function curve also effectively reflects the algorithm’s performance. Generally, a lower loss-function value indicates that model predictions are closer to true values, suggesting better model performance. Figure 8 also intuitively shows that the accuracy improves with increasing iterations. This demonstrates the model’s high accuracy and reliability in fault detection. The model training duration was 1924.37 s (32.07 min), and the peak GPU memory usage during training was 6553.6 MB (6.4 GB), indicating that this method maintains efficient training while requiring relatively low hardware resources, which is beneficial for the practical deployment of future diagnostic engineering.

This study builds six diagnostic models, the CNN-LSTM, CNN-BiLSTM, CNN-xLSTM, Transformer-LSTM, Transformer-xLSTM, and Transformer-BiLSTM models.

**Fig. 9:**
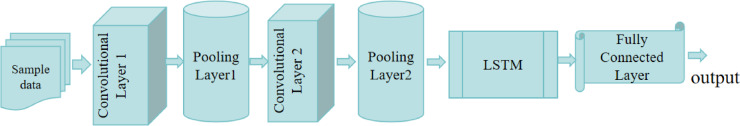
The architecture diagram of the CNN-LSTM model.

Transformer-LSTM, Transformer-GRU and Transformer-BiLSTM adopt the same structure and hyperparameters as the main method model, only replacing the LSTM module. The architecture of the CNN-LSTM model is illustrated in the following Fig. 9. The CNN component consists of two convolutional layers designed to capture spatial features from time-series data. Each convolutional layer is followed by a max pooling layer to enhance the network’s stability and nonlinear representation capability. Based on the CNN features, an LSTM layer is introduced to model long-term temporal dependencies, followed by a fully connected layer with a 0.4 dropout rate for classification. The model parameters are as follows: the first convolutional layer has a depth of 32 and a kernel size of 3 × 1, and the second has a depth of 64 and the same kernel size of 3 × 1, both using the ReLU activation function. To conduct comparative experiments, the LSTM layer is replaced with GRU, BiLSTM, and xLSTM variants, resulting in the CNN-GRU, CNN-BiLSTM, and CNN-xLSTM models, respectively.

**Fig. 10:**
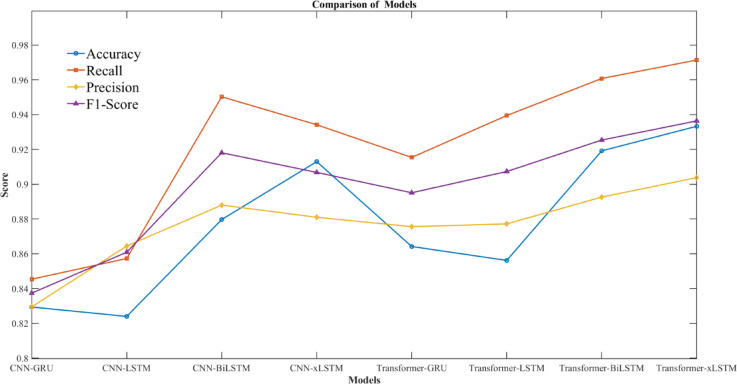
Comparison chart of model experimental results.

Which share the same dataset and parameters. These models are trained to verify the effectiveness of the proposed method.Comparison effect as shown in Table 6; Figs. 10.

**Table 6:** Comparison of model experimental results.

Model	Accuracy	Recall	Precision	F1-score
CNN-GRU	0.8294	0.8454	0.8295	0.8374
CNN-LSTM	0.824	0.8573	0.8645	0.8609
CNN-BiLSTM	0.8797	0.9503	0.888	0.9181
CNN-xLSTM	0.913	0.9342	0.881	0.9068
Transformer-GRU	0.8642	0.9154	0.8756	0.8951
Transformer-LSTM	0.8562	0.9395	0.8772	0.9073
Transformer-BiLSTM	0.9192	0.9608	0.8926	0.9254
Transformer-xLSTM	0.9333	0.9714	0.9038	0.9364

From the experimental results, the Transformer-xLSTM model achieves 93.33% accuracy, 97.14% precision, 90.38% recall, and 93.56% F1 score, surpassing other models. This further confirms its superiority and reliability in diagnosing faults in time-series data.

To more intuitively demonstrate the superior performance of the combined model, we conducted a comparison between the Transformer-xLSTM model and the two separate individual models. The results are shown in Table 7 .

**Table 7:** Independent model comparison.

Model	Accuracy	Recall	Precision	F1-score
Transformer-xLSTM	0.9333	0.9714	0.9038	0.9356
xLSTM	0.852	0.9309	0.8239	0.8742
Transformer	0.8369	0.852	0.877	0.832

Experimental results demonstrate that the Transformer-xLSTM model outperforms individual Transformer and xLSTM models across all evaluation metrics. It can identify faults more accurately, offering a more reliable basis for diagnosing real-world engine power-loss faults.

### Conclusion

 By extracting and converting key real-world user-data into data resembling that from on-site test-driving, the classification-based method for diagnosing engine power insufficiency was verified. Results show that this method can accurately identify such faults remotely. It offers a more convenient and efficient solution for commercial vehicle maintenance and strong support for vehicle operation management.

## Discussion

For the diagnosis of insufficient engine power in commercial vehicles, traditional methods have relied on on-site test drives, which lead to significant resource consumption and are subject to human bias. This study proposes a data-driven diagnostic framework to address these issues. By analyzing real-time driving data, it was found that vehicle speed, acceleration, and the rate of change of throttle position are key indicators for detecting insufficient engine power. The rate of throttle change was identified as a reliable indicator for detecting the driver’s intention to accelerate, revealing different interaction patterns between the driver and the vehicle under normal power and insufficient power conditions. By segmenting acceleration intent based on the rate of throttle change and modeling each segment separately, the model can more accurately identify anomalies under different operating conditions, revealing the dynamic relationship between power failure and driving behavior. Additionally, a separate deep learning framework was established for diagnosing data with missing acceleration intent to achieve comprehensive diagnosis of faulty data. This approach shifts the evaluation method from traditional diagnostics, which focus solely on mechanical indicators, to a performance assessment led by artificial intelligence. Therefore, the proposed framework provides a feasible path for remote and online diagnosis that aligns with real driving experiences.

To improve this method, we can expand the dataset to boost the integrity of technical-solution verification. Meanwhile, we can apply ensemble learning to integrate the two methods, achieving comprehensive diagnostic coverage.

## Data Availability

The datasets analyzed in the current study are available from the corresponding author upon reasonable request.
